# Cervical Mesonephric Adenocarcinoma: A Case Report of a Rare Gynecological Tumor from Embryological Remains of the Female Genital Tract

**DOI:** 10.1055/s-0041-1725051

**Published:** 2021-03-30

**Authors:** Catarina Reis-de-Carvalho, Carolina Vaz-de-Macedo, Santiago Ortiz, Anabela Colaço, Carlos Calhaz-Jorge

**Affiliations:** 1 Department of Obstetrics, Gynecology and Reproductive Medicine, Centro Hospitalar Universitário Lisboa Norte, Lisbon, Portugal; 2 Department of Gynecology and Obstetrics, Hospital Garcia de Orta, Lisbon, Portugal; 3 Department of Pathology, Centro Hospitalar Universitário Lisboa Norte, Lisbon, Portugal

**Keywords:** cervix, embryonic remnants, neoplasia

## Abstract

**Introduction** Malignant mesonephric tumors are uncommon in the female genital tract, and they are usually located where embryonic remnants of Wolffian ducts are detected, such as the uterine cervix. The information about these tumors, their treatment protocol, and prognosis are scarce.

**Case report** A 60-year-old woman with postmenopausal vaginal bleeding was initially diagnosed with endometrial carcinoma. After suspicion co-testing, the patient underwent a loop electrosurgical excision of the cervix and was eventually diagnosed with mesonephric adenocarcinoma. She was subjected to a radical hysterectomy, which revealed International Federation of Gynecology and Obstetrics (FIGO) IB1 stage, and adjuvant radiotherapy. The follow-up showed no evidence of recurrence after 60 months.

**Conclusion** We present the case of a woman with cervical mesonephric adenocarcinoma. When compared with the literature, this case had the longest clinical follow-up without evidence of recurrence, which reinforces the concept that these tumors are associated with a favorable prognosis if managed according to the guidelines defined for the treatment of patients with cervical adenocarcinomas. Though a rare entity, it should be kept in mind as a differential diagnosis for other cervical cancers.

## Introduction

Dur ing embryogenesis, the mesonephric (Wolffian) ducts connect the primitive kidney (mesonephros) to the cloaca and run parallel to the Mullerian ducts. 1 In a female fetus, the Mullerian structures develop into the fallopian tubes, uterus, cervix, and upper third of the vagina, but the mesonephric ducts regress as female development progresses. Mesonephric remnants can be detected in the lateral wall of the vagina and uterus, the broad ligament, the mesosalpinx, and ovarian hilus with a frequency of about 20%. 2 Malignant mesonephric tumors of the female genital tract are uncommon and are found in sites where there are embryonic remnants of Wolffian ducts. 3

The correct identification of mesonephric lesions is imperative for the proper management of the patient. 2 At present, there is no consensus on a standardized treatment protocol for malignant mesonephric cervical tumors. 4

In the present case, a 60-year-old woman was initially referred to an oncology center with a diagnosis of endometrial adenocarcinoma, and, after clinical, imaging, and laboratory reassessment, the exact diagnosis of a cervical mesonephric adenocarcinoma was reached. Only a high index of suspicion allowed the correct diagnosis and, thus, a favorable outcome.

## Case Report

A 60-year-old white woman, obese (body mass index [BMI] = 31 kg/m2), was referred to a Portuguese tertiary hospital from an African country, with the diagnosis of undifferentiated endometrial carcinoma with endocervical involvement. The diagnosis was established after an endometrial curettage in the context of a single episode of a moderate postmenopausal vaginal bleeding, 4 months before, that did not require transfusion blood support. The patient did not report weight loss or abdominal pain. The hospital where the patient was initially seen was not able to perform endoscopic or imaging techniques, so, according to a health cooperation protocol between Cabo Verde and Portugal, she was referred to Portugal to proceed with the investigation and treatment. In Portugal, the patient's physical observation revealed a good general health status, globous and soft abdomen, external genitals and vagina with moderate atrophy, no blood collected in the vaginal canal, and a cervix flattened and without visible macroscopic lesions. On bimanual palpation, the cervix was increased in consistency, mobile, the uterus was small, and the adnexa were not palpable. On rectal examination, the parametrium was free on both sides. Suspicious adenopathies were not evident.

A pathological review of the slides of the biopsies previously performed confirmed the presence of fragments of the endometrium, myometrium, and endocervical mucosa with infiltration by malignant neoplasia tissue with an inconclusive histological type.

The patient underwent a transvaginal ultrasound that revealed a normal-sized uterus with an endometrial thickness of 5.5 mm and no adnexal findings.

The hysteroscopy showed an endometrium white and smooth compatible with atrophy, without macroscopic lesions, and there was no need to perform additional biopsies.

Based on these contradictory findings, we proceeded with our investigation performing a co-testing of the cervix, which revealed a high-grade squamous intraepithelial lesion and human papillomavirus (HPV) 16. Tumor markers (CEA, CA-125, and SCC) were negative. We performed a colposcopy, which was unsatisfactory because of a transformation zone type 3, followed by loop electrosurgical cervical excision procedure (LEEP). The pathological examination of the excised part of the cervix revealed the presence of invasive endocervical adenocarcinoma in the upper segment of the specimen, with positive surgical margins. Pelvic magnetic resonance imaging and toraco-abdominal tomography showed a cervical mass measuring 13 × 11 × 7 mm with stromal invasion and no signs of parametrial or lymphatic invasion.

The patient underwent a nerve-sparing radical hysterectomy with dissection of the parametrium to the medial aspect of the internal iliac artery and vein and a vaginal margin of 22 mm (type C of Querleu-Morrow classification), bilateral salpingo-oophorectomy, and pelvic lymphadenectomy. No invasion or adhesions to adjacent organs were grossly apparent.

The pathology observation showed a tumor with 15 × 12.5 × 7 mm, deep stromal invasion of 6 mm, extensive angiolymphatic invasion, and a distance from the invasive carcinoma to the radial (circumferential) margin of less than 4 mm. The parametria, the vaginal cuff margin, and the 28 lymph nodes excised were tumor-free.

**Fig. 1:**
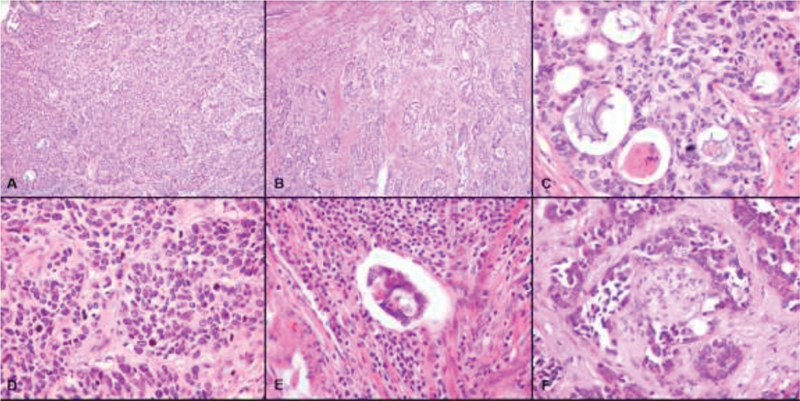
Anatomopathological examination of the tumor (Histologically, the tumor showed a variety of architectural patterns, including solid [A - H&E x100] and tubular glands that invaded the deep cervical stroma in a haphazard growth [B - H&E x100]). These glands are lined by atypical mucin-free cuboidal epithelium cells containing eosinophilic secretion in their lumina (C - H&E x400). There is elevated mitotic activity (D - H&E x400), and images of lymphatic (E - H&E x400) and perineural invasion (F - H&E x400).

The microscopic examination of the tumor described infiltrating tubular structures, lined by cuboidal epithelium exhibiting mild-to-moderate nuclear atypia and containing luminal eosinophilic and hyaline secretion ( Fig. 1 ). The proliferation of mesonephric tubules and ducts arranged in lobules was seen adjacent to the primary tumor. The tumor was positive for CK8-18, p16, CEA, and calretinin but negative for CK5/6, CK14, vimentin, CK5-6, inhibin, and both estrogen and progesterone receptors. Based on these findings, the diagnosis of mesonephric adenocarcinoma arising in mesonephric hyperplasia was established, staged at IB1 (International Federation of Gynecology and Obstetrics [FIGO] staging system for cervical cancer 2018).

The patient underwent adjuvant pelvic teletherapy (40 Gray [Gy] of intensity-modulated radiation therapy [IMRT] in 20 fractions of 2 Gy daily), brachytherapy (12 Gy with the dose rate of 2.4Gy/h for a period of 2 days with a 1-week interval) and concurrent chemotherapy (5 weekly treatment sessions of cisplatin at a dose of 35 mg/m ^2^ ) with good tolerance.

The patient maintained clinical surveillance according to national protocols (every 4 months in the 1 ^st^ year, every 6 months in the 2 ^nd^ year and annually from the 3 ^rd^ to the 5 ^th^ year) with annual cytology. No further diagnostic tests were necessary. She has remained disease-free up until the 60-month follow-up visit.

## Discussion

Mesonephric adenocarcinoma, one of the rarest tumors of the female genital tract, is defined by the World Health Organization (WHO) as a tumor with tubular glands lined by mucin-free cuboidal epithelium, luminal eosinophilic hyaline secretions with solid papillary, ductal or retiform architectural arrangements deriving from remnants of mesonephric (Wolffian) ducts. 5 These ducts run along the Mullerian ducts. In the female, the Wolffian ducts eventually regress without the presence of testosterone stimulus. Still, vestigial mesonephric remnants can be found in the meso-ovaries, the broad ligament, the lateral walls of the cervix, and rarely the vagina and corpus uterus. 6 They are not uncommonly encountered deep in the cervix's lateral walls (up to 22% of cases), where they give rise to hyperplasia but, rarely, malignant mesonephric tumors. 7 The most common clinical presentation is abnormal vaginal bleeding. Our patient displayed similarities with previously published cases in terms of age and symptoms at presentation and early stage of the disease. 8

Distinguishing risk factors have not yet emerged for the development of mesonephric lesions and carcinoma. 3 The association of these unusual adenocarcinomas with HPV infection is unclear. 4 While the association of usual adenocarcinoma with HPV infection is clear and well established in the variants of rare adenocarcinomas, such as clear cell, serous, and mesonephric, the rate of detection of DNA HPV is low or even absent. 9 10 However, few reports of HPV testing in rare adenocarcinoma variants are available, and the results are inconsistent. In reported cases, mesonephric adenocarcinoma is one of the few subtypes of cervical cancer that is not related to HPV. 11 However, in this case, one of the discriminating characteristics is the fact that HPV DNA has been identified. The pathological analysis of the tumor performed in this case corroborates the cytopathic effect of HPV, namely through the presence of koilocytosis and p16 positive staining. Nevertheless, p16 overexpression into these tumors is rare and may not correlate with HPV status. 5 12 We are unable to explain the association identified, but we hypothesized that it can be a non-causal association or that it may be a usual type of endocervical adenocarcinoma with a malignant transformation of the mesonephric component present in the cervix.

The differential diagnosis of mesonephric neoplasm is challenging. The main point at issue is to differentiate malignant mesonephric adenocarcinoma and clear-cell carcinomas. 6 The differential diagnoses also include other tumors that have no relation to the mesonephric system, such as yolk-sac tumor, endometrial stromal tumor, malignant Mullerian tumor, carcinosarcoma, female adnexal tumor of probable Wolffian origin (FATWO), and sex cord-stromal tumor. 10 13 Benign proliferative mesonephric lesions include a scope of symptomatic lesions associated to a diffuse hyperplastic process that may have a deeply infiltrative appearance, like hyperplasia of mesonephric remnants. 7 13 They are unusual conditions that are the common origin of mesonephric carcinomas, and demand a differential diagnosis from it. 7 13

The cytological and architectural features are the base for the mesonephric adenocarcinoma diagnosis. For this reason, it is fundamental that the clinical approach allows for obtaining tumor biopsies. However, the diagnosis can be challenging, especially on biopsy materials and frozen sections. 10 This fact explains why our case initially had an incorrect diagnosis of endometrial adenocarcinoma and underlines the importance of performing a diagnostic LEEP of the cervix in cases of suspected cervical neoplasia.

The histological patterns of the mesonephric adenocarcinomas are multiple, as they usually exhibit a mixture of morphologic characteristics. 5 In contrast to mesonephric hyperplasia, the typical background lesion of a mesonephric carcinoma does not have a lobular architecture, the nuclei appear cytological malignant, 11 and the tubular glands are lined by mucin-free cuboidal epithelium containing luminal eosinophilic hyaline secretions, with solid, papillary, ductal, or retiform architectural arrangements with different grades of atipia. 4 5 13

Studies report mesonephric carcinomas with neuroendocrine, 14 sarcomatous, 15 16 and low-grade serous component. 17 One study revealed that up to 25% of tumors of mesonephric origin could have a sarcomatous element. 13 Molecular studies have shown that these different components share similar genetic mutations and are usually associated with a worse prognosis. 10

In light of the difficult diagnosis, immunohistochemistry helps to point towards other neoplasms. However, there is no specific immunohistochemistry profile established for mesonephric tumors. 2 Positive immunostaining for CD10, CK7, calretinin, epithelial membrane antigen (EMA), PAX8, CA125, and vimentin along with negative immunostaining for CEA and EP/PR is suggestive of a mesonephric origin. 9 15 Recently, GATA3 was shown to be a highly specific and sensitive marker to distinguish mesonephric origin from other carcinomas of the gynecologic tract. 18 The combination of GATA3, PAX8, and CD10 positivity is common in this type of carcinoma. 16

The tumor reported in the present case showed an immunoreaction pattern consistent with a previous description ( Chart 1 ).

**Chart 1:** Comparison of the immunohistochemical results of mesonephric adenocarcinoma between the present case and the literature

Immunohistochemical Biomarker	Case	Literature 19
CK 20	−	−
EMA	+	+
Calretinin	+	+
Vimentin	−	+
Estrogen receptors	−	−
Progesterone receptors CEA	−	−
CD 10	+	−
GATA3	+	NP
Pancytokeratin	+	+
CK 5–6	NP	+
P16	+	−

**Abbreviations** : HPV, human papillomavirus; LEEP, loop electrosurgical excision procedure; NP, Not performed.

Most of the patients previously reported having mesonephric adenocarcinomas were, similarly to our patient, diagnosed at an early stage and treated with radical surgery and (neo-)adjuvant chemo- or radiotherapy, depending on the disease staging. 5 Considering the lack of evidence, it seems reasonable to manage patients according to the current guidelines for cervical adenocarcinoma of a similar stage. 7 Prognosis cannot be accurately predicted due to the small number of cases with long enough follow-up. 4 Importantly, controversy exists in the natural history of malignant mesonephric tumors. Zhang et al. 13 concluded that most patients with uterine mesonephric adenocarcinomas were reported to be disease-free during the short follow-up period. Dierickx et al. 11 reported that patients with a stage I mesonephric adenocarcinoma had a mean recurrence interval of 24 months, and 4 out of 24 of these patients with adequate follow-up died. 11 The same authors emphasized that mesonephric adenocarcinomas seem to have a worse prognosis than squamous-cell carcinoma and usual type adenocarcinoma. 11 For cervical mesonephric adenocarcinomas, prior studies have shown that the rate of 10-yr survival is only 30%, and frequent recurrences and pelvic metastasis characterize the clinical course. 8 10

Among the cases described in the literature, the highest average survival time is 50 months. 5 Our case stands out by presenting the longest disease-free time reported (60 months), offering new insights that can help in counseling and guiding these cases.

## Conclusion

Even though it is a rare entity, mesonephric adenocarcinoma should be kept in mind as a differential diagnosis for cervical cancer. The present case stands out from various angles: the rarity of the diagnosis, the difficulty in obtaining tumor specimens, and the association with HPV. Because cytological, architectural, and immunohistochemical features are the base for the diagnosis, it is fundamental that the clinical approach includes tumor biopsies for correct classification. We should manage them according to the guidelines for cervical adenocarcinomas and expect a favorable long-term prognosis in early-stage disease.
